# Feeding intensity and molecular prey identification of the common long-armed octopus, *Octopus minor* (Mollusca: Octopodidae) in the wild

**DOI:** 10.1371/journal.pone.0220482

**Published:** 2020-01-27

**Authors:** Qi-Kang Bo, Xiao-Dong Zheng, Zhi-Wei Chen

**Affiliations:** 1 Institute of Evolution and Marine Biodiversity, Ocean University of China, Qingdao, China; 2 Breeding station of Bohai Sea aquatic resources, Tianjin Fisheries Research Institute, Tianjin, China; 3 Key Laboratory of Mariculture, Ministry of Education, Ocean University of China, Qingdao, China; University of Sydney, AUSTRALIA

## Abstract

The common long-armed octopus, *Octopus minor*, is an important component of systems and supports the local fisheries in the coastal areas of northern China. For the fishery management and artificial breeding, especially for the management of exclusive conservation reserves, its role in the ecosystem requires assessment. Therefore, the feeding intensity of *O*. *minor* was studied from April to July 2014 when females reaching ovary maturation, and prey composition was identified from stomach contents using a DNA barcoding method. Of the 172 sampled octopuses, 66 had stomach contents that were nearly digested into pulp. On the whole, the feeding intensity of octopus remained more or less the same during the first three months and significantly decreased in July. The changes of feeding intensity were different between females and males; in females, the intensity of feeding decreased from April to July; in case of males, however, the feeding activity increased from April to June and decreased thereafter. The feeding intensity of the females was extremely greater than that of the males. *O*. *minor* was a generalist predator and based on homology searches and phylogenetic analysis, a total of 10 different taxa were identified in the stomach contents. In terms of percent composition by frequency of occurrences (%N), fishes accounted for the most of the octopuses diet (50%), followed by cephalopod (25%), crustaceans (21.7%), annelid (1.7%) and nematode (1.7%). The families of Gobiidae and Octopodidae appeared in all months and Protunidae appeared in three months. The results confirmed that Gobiidae family (45.8%, by frequency of occurrences) was an important source of food during the time when females reaching ovarian maturation. From April to July, the observed cannibalism showed an increasing trend. Controlling and reducing fishing production of Gobiidae fishes in conservation area are recommended from April to June when female octopuses are actively feeding.

## Introduction

**Fig 1:**
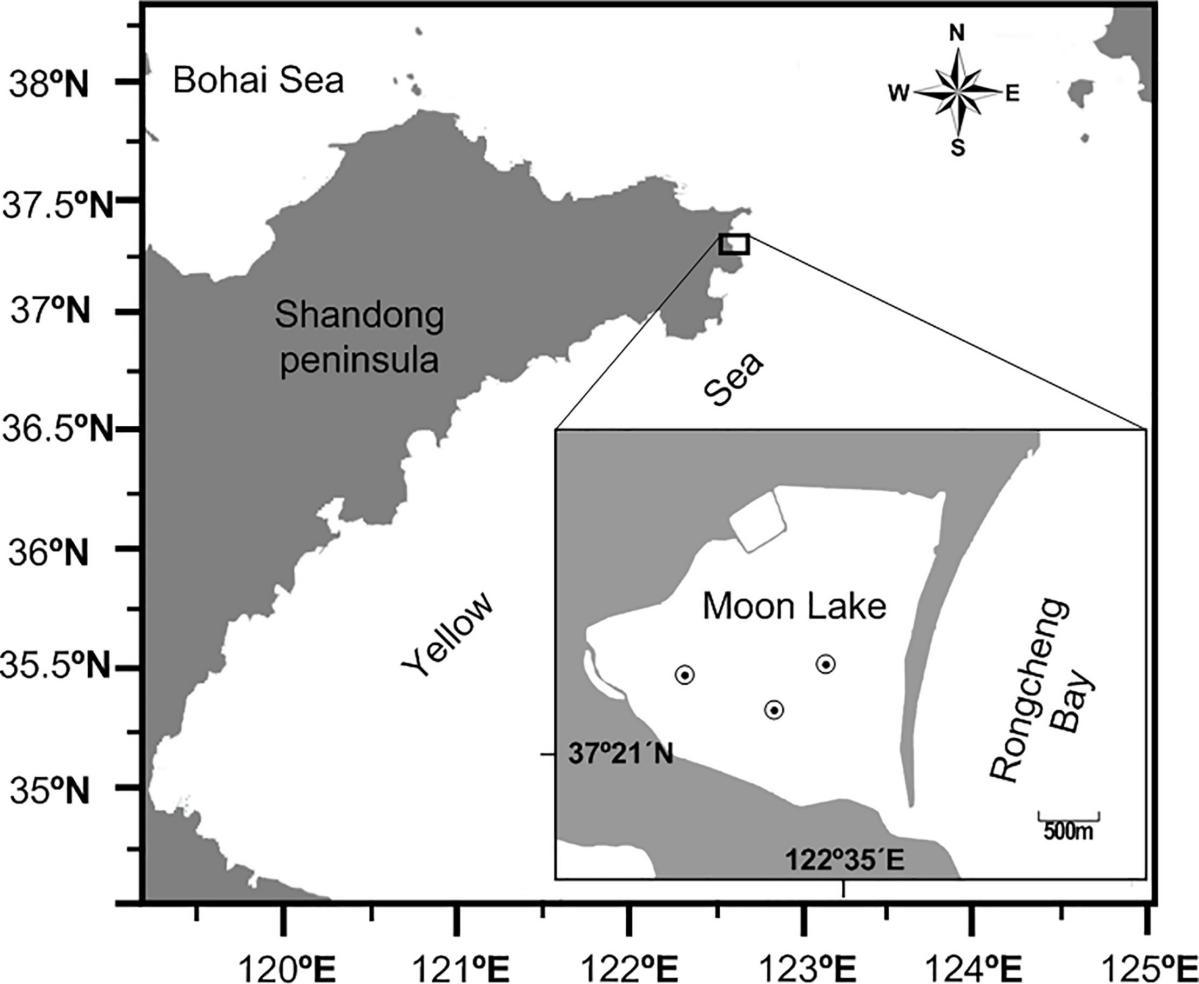
The location of the sampling site at the Moon Lake and the dots show the sampling sites.

The common long-armed octopus, *Octopus minor* (Sasaki 1920) is a benthic and neritic octopod that has become a commercially important species in the north of China and in South Korea [1, 2] and is identified to be furthermore ecologically important as a generalist predator. A national germplasm reserve was established at the Moon Lake (Fig 1) in 2012 to serve as conservation for the genetic resource of *O*. *minor* because of the overfishing. Moon Lake is a shallow (depth <3 m) lagoon with a muddy, bivalve-covered bottom, and patches of sea grass growing in the subtidal zone, which provides a good habitat for *O*. *minor*. Additionally, a program of artificial propagation and release of *O*. *minor* has been launched in the north of China [2].

The five methods used to assess the natural feeding habits of marine animals include the examination of prey remains from middens [3], direct observation [4, 5], stomach content analysis [6, 7], isotopic assessment [8], trophic tracing [9] and molecular prey identification [10–12]. In some studies, several different methods have been used in combination [11, 13, 14].

A range of factors have made it difficult to determine the natural diet of *O*. *minor*. The primary challenge is that the oesophageal diameter of the octopus is physically limited as it passes through the brain, therefore, the octopus’ beak bites small pieces of tissue to swallow, avoiding the ingestion of hard skeletal material [11], and it is difficult to make an accurate identification of prey with lack of hard skeletal material. Moreover, middens are difficult to be collected on a large scale. *O*. *minor* burrows deep (0.3–0.6m) interconnecting tunnels as nest in muddy marine bottom, leaving only digging-holes and breathing-holes on the surface, into which it hides itself [15]. Their prey remains are generally left underground. Even if the prey remains were pushed out of the nests, the light material would be easily removed by biotic and abiotic factors, while the heavier material would be buried under the mud. Rapid digestion rates [16] and external ingestion [17] make the stomach contents visually unidentifiable. These specialized feeding strategies tend to bias the data on prey species when morphological analysis is used.

Food is an important factor to the extent that it governs growth, fecundity and migratory movements. An understanding of the relationship between octopus species and their food items helps to locate potential feeding grounds, which may, in turn, be helpful for the exploitation of these resources. The extent of variation in feeding must be taken into account when studying the diet of a generalist predator. The species, size and type of their diet in the wild are often dominated by many factors such as gonadal maturity stage, seasonal variation [18], the body size which reflects their preying ability [3, 14] and benthic assemblages which determine diet availability [19]. In addition, what should be taken into account is that octopods exhibit strong dietary preferences when given the same opportunity to different diets items [20, 21–24].

Few studies have focused on the ecology of *O*. *minor*, whose diet is roughly known to include crustaceans, molluscs, polychaetes and fish [25, 26]. Investigations into the natural diet of this species during gonadal maturation, are of more research value, and can provide trophic relationship information for fishery management, especially for the management of exclusive conservation reserves. It also provides a prey reference for aquaculture and artificial breeding of this commercially important species. In this study, we applied the method of molecular prey identification, DNA barcoding method, to identify the natural prey of *O*. *minor* based on stomach contents.

## Materials and methods

### Specimen collection

All the analyses have been carried out using freshly dead specimens collected from local fishermen. No use of live animals has been required for this study and no specific permissions were needed for the sampling activities in all of the investigated areas because our species of interest is commercially harvested (not endangered nor protected) and it was caught in areas where fishing is allowed. One hundred and seventy-two adult *O*. *minor* specimens were captured using cage nets and the nets were 7m long and were placed, one net in each sampling site, in the east-west direction during the evenings and collected at dawn from Moon Lake (122°35’E, 37°21’N, Fig 1) from April to July 2014. The same number of males and females were collected each month.

For the stomach contents analysis, all specimens were brought to the laboratory where their stomach contents were removed. Each stomach was opened and the contents were flushed into cryogenic vials. To avoid potential contaminants (e.g., blood and tissue attached to the stomach from the predator), the exterior surface of each stomach was washed with sterile, distilled water before removing the stomach contents [27]. The stomach contents, potential residue of prey, were weighed and then preserved in 70% ethanol at -20°C for later DNA analysis [28]. Stomach content samples were numbered monthly, AP01 to AP19, MA01 to MA13, JN01 to JN19 and JL01 to JL15 from April to July, respectively. The dorsal mantle length (DML), the distance between the posterior midpoint of the mantle and the midpoint of the eyes, and wet body weight (W) were also measured. Feeding intensity during the study months was determined based on the degree of fullness of the stomach and the condition of feeding activity was determined from observations of the degree of stomach distension as described by Pillay (1952) [29]. Octopuses with stomachs that were gorged, full, ¾full, ½full were considered to have been actively feeding (AF), while stomach ¼full, little and empty were considered to denote poor feeding activity (PAF). The percentage of octopus in the AF and PAF condition in both sexes for each month was calculated.

### DNA extraction and sequence acquisition

Before the DNA extraction, stomach contents were evenly ground in a homogenizer. Duplicate 20–40 mg samples of mixed tissue items were subsampled from each stomach contents and genomic DNA was then isolated by standard phenol-chloroform purification procedures. Species identity of preys in stomach contents was confirmed by sequencing the mitochondrial cytochrome oxidase Ⅰgene (COⅠ) and a fragment of COⅠwas amplified using the universal primers [30].

Each polymerase chain reaction (PCR) was carried out in 50-μL volumes containing 2U Taq DNA polymerase (Takara Co.), about 60 ng template DNA, 0.2 mM dNTPs, 0.25 μM of each primer, 2 mM MgCl_2_ and 1×PCR buffer. The PCR amplification was performed on a GeneAmp® 9700 PCR System (Applied Biosystems). Cycling conditions consisted of an initial denaturation at 94°C for 3 min, followed by 35 cycles of: denaturation at 94°C for 1 min, annealing at 50°C for 1 min, extension at 72°C for 1 min, and a final step of 5 min at 72°C.

Amplification products were confirmed by 1.5% TBE agarose gel electrophoresis stained with ethidium bromide. The cleaned product was prepared for sequencing using the BigDye Terminator Cycle Sequencing Kit (ver.3.1, Applied Biosystems) and sequenced bidirectionally using an ABI PRISM 3730 (Applied Biosystems) automatic sequencer. PCR products producing multiple bands indicated that more than one prey species were present. Those PCR products were cloned using the TOPO TA Cloning Kit (Invitrogen). Eight colonies per sample were selected for colony PCR amplification and sequencing using the primers M13 (forward): GTAAAACGACGGCCAG, andM13 (reverse): CAGGAAACAGCTATGAC.

### DNA analyses and statistical analysis

Before the homology searches, sequences of COⅠ were assembled and edited separately using DNASTAR software (DNASTAR, Inc.), and then aligned with CLUSTAL_X 1.81 using the default settings [31]. Sequences were considered to be part of the same ‘‘operational taxonomic unit” (OTU), if there was less than a 1% sequence divergence, allowing for the intraspecific variation and Taq polymerase errors [11, 13].

Taxon identification was made by the homology searches. All of the obtained sequences were submitted and identified using the Identification System (IDS) in the Barcode of Life Database (BOLD, www.boldsystems.org) and the Basic Local Alignment Search Tool (BLAST) query algorithm in GenBank to establish whenever possible the identification of the ingested material. For the phylogenetic analysis, the maximum likelihood (ML) method was chosen to infer evolutionary history. Bootstrap probabilities with 1,000 replications were calculated to assess reliability on each node of the ML tree. Sequence divergence calculation and evolutionary analyses were conducted in MEGA 6 software based on the Kimura 2-parameter model (K2P) [32]. The ML tree contained all of the sequences obtained from the stomach contents, together with the closest matches that were downloaded from BOLD databases and GenBank. The criteria to assign identification to the species level required the sequence similarity display >98% in the BOLD database or BLAST [11] and, if not, identification was restricted to the highest taxonomic lineage supported by bootstrap probabilities higher than 70% in the consensus tree [14, 15]. Suitability test of different distribution proportion of the AF and PAF between sexes and among months were verified by the Chi-squared fit test (χ2). Statistical analyses were performed using IBM SPSS statistics version 20 (IBM, Chicago, IL, USA). To corroborate that the number of analyzed stomachs was adequate for diet description, a cumulative prey curve was generated using the Estimate S Version 8.2 based on the prey identified [33]. The number of samples was assumed to be sufficient to describe the diet when the curve approaches the asymptote.

## Results

### Feeding intensity

**Fig 2:**
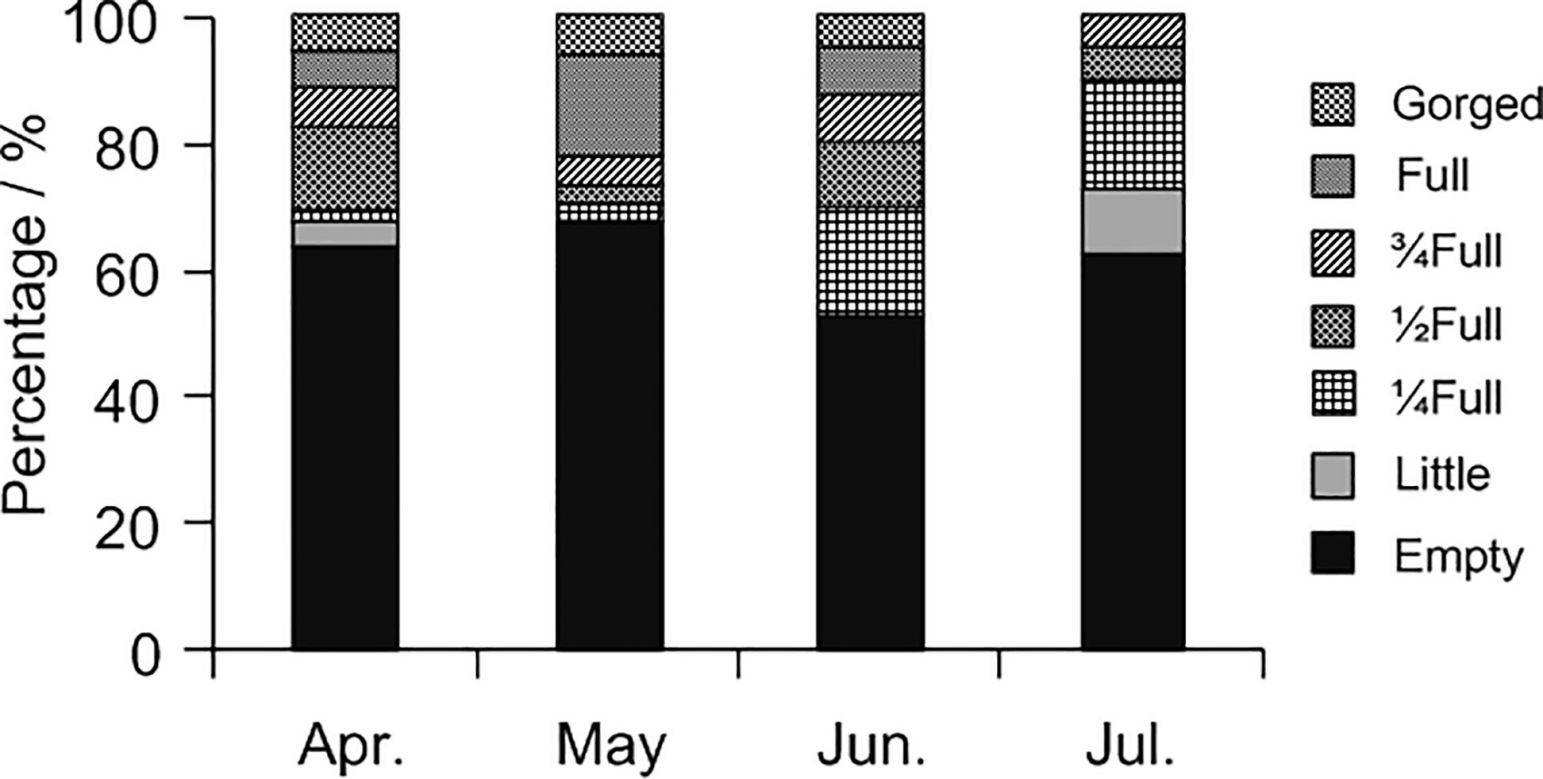
The distribution of stomachs with different degree of distension for *Octopus minor* in each month from April to July.

172 adult octopus individuals were obtained and the monthly DML (mean±SD) ranged from 8.68±0.95 to 10.55±1.14 cm, the W (mean±SD) ranging from 120.47±24.99 to 202.80±42.66 g. The distribution of stomach with different degree of distension from April to July was presented in Fig 2. Of the 172 octopus individuals, 66 had contents in their stomachs. Feeding intensity of *O*. *minor* varied to an extent in respect to the months, and more than half of the stomachs were found to be empty (Fig 2). On the whole, the feeding intensity of octopuses remained more or less same during the first three months (p>0.05) and significantly decreased in July (p<0.05). However, the changes of feeding intensity were different between females and males. The AF percentage of the females generally decreased, showing a decrease in the intensity of feeding for females. In case of the males, the feeding activity increased from April to June and decreased thereafter. The feeding intensity of the females was extremely greater than that of the males except the month of June (Table 1).The maximum feeding intensity occurred in April for females and in June for males respectively. Although feeding intensity recovered in June, the feeding intensity of the males kept at a low level (25%, percentage of AF) that was just slightly higher than the minimum of the females (20%).

**Table 1:** The percentage of octopus with active feeding (AF) activity in the whole population, females and males in each month from April to July, including level of significance of difference between sexes by Chi square fit test. Data in the same column having different superscripted letters are significantly different (p<0.05).

Month	Percentage of AF (%)			
	Population	Female	Male	Level of significance
Apr.	30.8^a^	50^a^	11.5 ^bc^	p = 0.000, **
May	29.5^a^	45^ab^	15 ^ab^	p = 0.000, **
Jun.	30^a^	35^b^	25 ^a^	p = 0.123, NS
Jul.	10^b^	20^c^	5 ^c^	p = 0.001, **

*, P<0.05

**, P<0.01; NS, not significant

### Molecular prey identification

**Fig 3:**
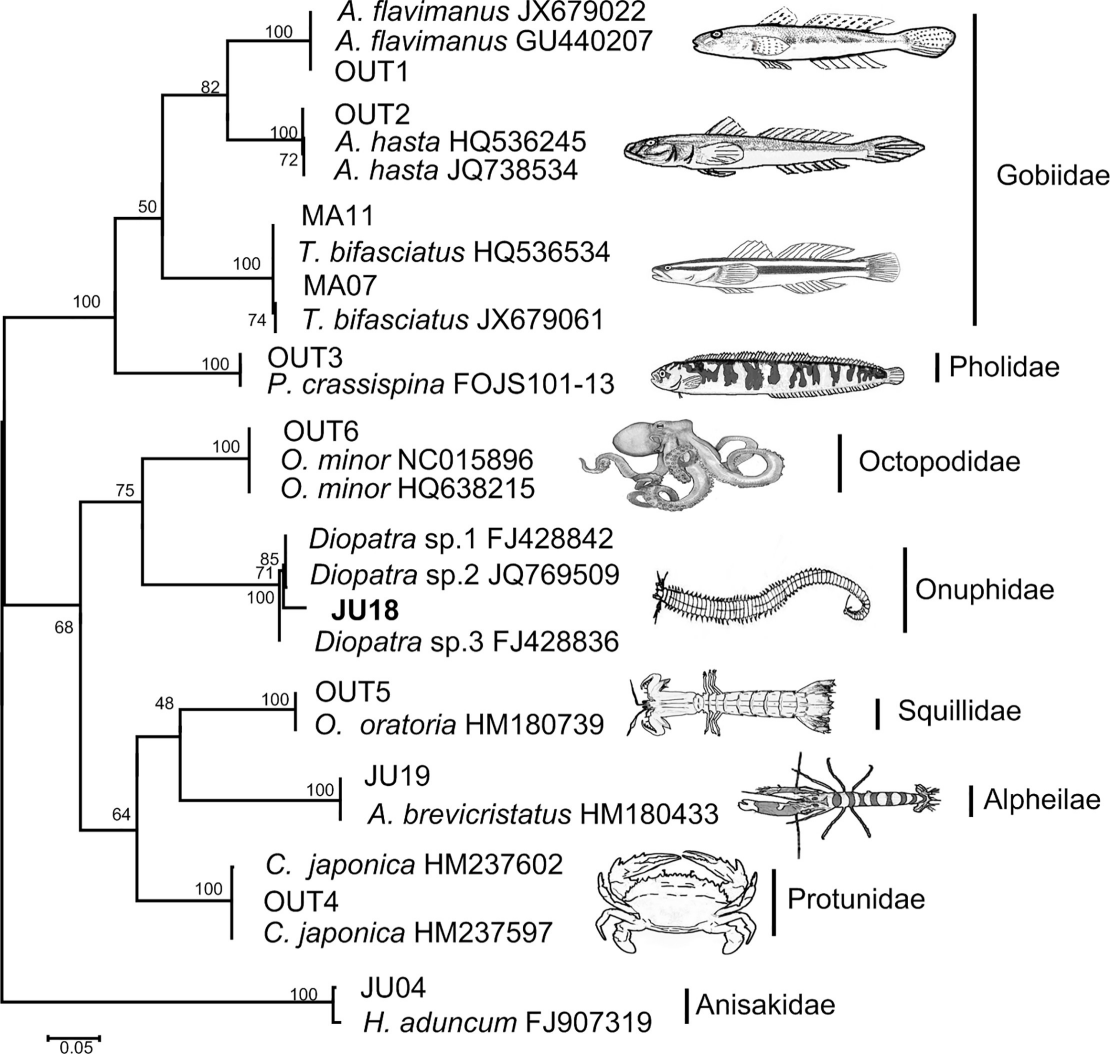
Maximum likelihood tree (ML tree) for all sequences obtained from stomach contents and the closest matches that were downloaded from BOLD databases and GenBank, that was conducted using MEGA 6 software based on K2p model.

All stomach contents were nearly digested into pulp and as a result, the prey items were impossible to visually identify. A total of 59 stomach contents yielded amplifiable DNA and 60 sequences were obtained, ranging from 500 bp to 708 bp (Table 2). All sequences were submitted to GenBank (Accession numbers MK688462-MK688521). Six OTUs were established, with a maximum sequence divergence of 0.1%. Of the 60 sequences, 59 clones showed similarities higher than 98% to reference sequences, allowing identification at species level. Only one clone displayed 97.56% similarity to reference sequences, and it was assigned to the *Diopatra* genus level (Fig 3). One stomach contained two kinds of prey, and the remaining 58 samples contained only one kind. The duplicate parallel PCR products from the contents of each stomach yielded the same sequences.

**Table 2:** Prey DNA detected in wild *O*. *minor* by cloning the COⅠ fragment gene, including min.-max. Length of sequences, GenBank Accession numbers and Sequence ID of closest matches, percentages of similarity obtained from BLAST and BOLD.

Order	Family	Species	Similarity %	Ac. Number or Seq. ID	Prey DNA	Min.-max. length of Seq.
Perciformes	Gobiidae	*Acanthogobius flavimanus*	99.85,100	JX679022, GU440207	OUT1	500–700
Perciformes	Gobiidae	*Acanthogobius hasta*	99.85,100	JQ738534, HQ536245	OUT2	640–697
Perciformes	Gobiidae	*Tridentiger bifasciatus*	100	JX679061, HQ536534	MA07, MA11	684
Perciformes	Pholidae	*Pholis crassispina*	100	FOJS101-13	OUT3	556–694
Decapoda	Protunidae	*Charybdis japonica*	100	HM237602, HM237597	OUT4	633–687
Decapoda	Alpheilae	*Alpheus brevicristatus*	100	HM180433	JU19	620
Stomatopoda	Squillidae	*Oratosquilla oratoria*	100	HM180739	OUT5	572–587
Eunicida	Onuphidae	*Diopatra* sp.	97.56	FJ428842	JU18	682
Ascaridida	Anisakidae	*Hysterothylacium aduncum*	98.91	FJ907319	JU04	679
Octopoda	Octopodidae	*Octopus minor*	99.66,100	HQ638215 NC015896	OUT6	562

*O*. *minor* is a generalist predator and a total of 10 different taxa were identified (Table 2). In terms of percent composition by frequency of occurrences (%N), fishes accounted for the most of the octopuses diet (50%), followed by cephalopod (25%), crustaceans (21.7%), annelid (1.7%) and nematode (1.7%). When considering the importance of prey to *O*. *minor* by number (%N, by frequency of occurrences), and ignoring the nematode that was parasite, the family with the highest %N were Gobiidae (45.8%), comprising *Acanthogobius flavimanus* (30.5%), *A*. *hasta*(11.9%) and *Tridentiger bifasciatus* (3.4%); followed by the Octopodidae (25.4%), comprising conspecifics, *O*. *minor*; Protunidae (13.6%), comprising one species *Charybdis japonica*; Squillidae (6.8%), comprising one species, *Oratosquilla oratoria*; Pholidae (5.1%), comprising one species *Pholis crassispina*; Alpheilae and Onuphidae, accounted for same percentage (1.7%), respectively, comprising one species each, *Alpheus brevicristatus* and *Diopatra* sp. Gobiidae and Octopodidae appeared in all months and Protunidae appeared in three months.

**Fig 4:**
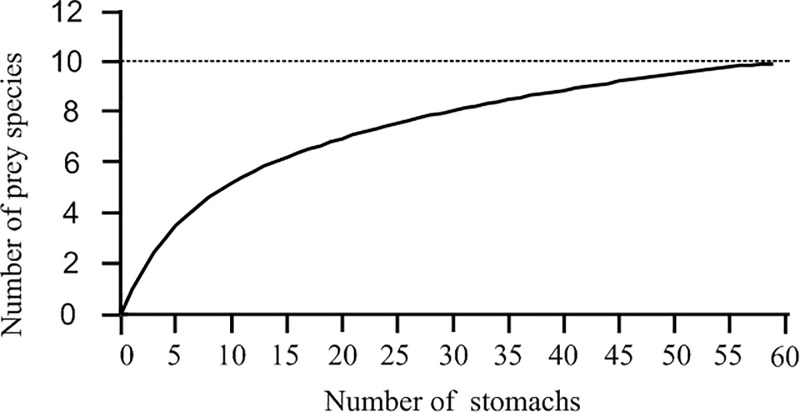
The species-accumulation cures.

The number of prey species consumed by *O*. *minor* in a month ranged 2 to 5, and in June the prey diversity was the most abundant, with 5 species. *A*. *flavimanus* and *C*. *japonica* appeared with the highest occurring frequency, followed by *A*. *hasta*. Cannibalism was found in 15 individuals, and cannibalism occurred 2, 3, 4 and 6 cases respectively from April to July, showing an increasing trend. And there was no difference in frequency of cannibalism between sexes (p>0.05). The cumulative prey curve approached the asymptote showing that 59 stomachs were adequate to describe the diet of this species (Fig 4).

## Discussion

Based on our recent studies, the male octopus reaches gonadal maturity in April, meanwhile, the females are at Ⅲ or Ⅳ stages (developing or maturing stages) of ovary development when the female octopuses begin egg production actively, which mainly involves the synthesis of vitellus. At these stages the female octopuses use energy directly from food, with no storage reserves being transferred from other organs to the gonads [34, 35]. Hence, it is unsurprising that the feeding intensity of the females was greater than that of the males. And the feeding intensity of the females decreased as synthesis of vitellus get done. In case of males, the feeding intensity was lowest in April possibly because they were engaged in mating [2, 36]. Although feeding intensity recovered in June, it kept at a low level that was just slightly higher than the minimum of the females, until a considerable feeding intensity reduction in July caused by the listlessness of the male after mating. Consequently, for the whole population, the feeding activity remained more or less the same during the first three months (p>0.05) with the interaction of changes in feeding activity of both sexes and considerably decreased in July.

The natural dietary of other similar octopuses has been studied using the morphological methods, like examining the prey remains from middens, direct observation and stomach content analysis, showing the most important prey species were crustaceans and mollusks with fishes being uncommon prey items in the diet. Rosas-Luis et al. (2019) showed that *O*. *insularis* most frequently consumed crustaceans, with the genera *Mithraculus* and *Etisus* being the most important in the diet [37]. For *O*. *vulguris*, collections of prey discards in octopus middens and in areas inhabited by octopuses revealed that molluscs comprise an estimated 80% of its diet [6] and from stomach contents, crustaceans were the most frequently found prey group in octopus stomachs, followed by mollusks [4, 14]. Grubert et al. (1999) showed *O*. *maorum* exhibited population specialization towards the crab, individual specialization on atherinid fishes [7]. Only in the stomachs of *O*. *mimus*, fishes were the second most frequently found prey group following the crustaceans [18]. However, the present study, firstly studying the dietary of *O*. *minor*, showed that fishes were the most frequently found prey group in stomachs (50.9%, frequency of occurrence), followed by itself (25.4%) and crustaceans (22%). The different dietary between *O*. *minor* and other similar octopuses reflected the different habit of this kind of octopus, and the diet of marine organisms is also affected by prey availability and the composition of marine life in the surrounding environment [14]. There were always a mass of unidentified prey items or a lot of prey items were assigned to higher taxon in previous studies using the morphological methods, making the biases inherent [3, 6, 7].

One advantage of molecular methods is that when morphological methods are ineffective, sufficient DNA can be recovered by successful DNA amplification [11, 12]. This can be achieved in poor quality samples as well because it requires only a small amount of tissue for DNA extraction. Moreover, Meusnier et al. (2008) have demonstrated that DNA barcoding could identify species with fragments as short as 100 bp with at least 90% efficiency [38]. The read lengths of the DNA fragments obtained in this study were 500–700 bp, and the relativly large read lengths strengthened the accuracy of identification. Only one clone was assigned to the genius level based on their supported topographical position on the bootstrap consensus tree (Fig 3).

One intriguing discovery we found was that almost all of the stomach contents (58 out of 59) contained only one kind of prey. In consideration of stochastic sampling errors when subsampling stomach contents for DNA extraction and lack of detection of some prey species arising from their low-concentration DNA to PCRs [13], duplicate subsamples with 20-40mg material from each stomach contents were used in this study. However, the same sequences were obtained from the duplicate subsamples showing that it was unlikely that the potential lack of detection of diet composition was due to the procedural errors. And the results also showed that although there distributed a large of mollusks, 15 molluscan species belonging to 14 families in Moon Lake [39], octopuses did not feed on mollusks except the conspecifics. However, the universal COⅠ primers used in this study are able to amplify COⅠ gene fragments from 11 invertebrate phyla, including mollusks as shown in previous studies [30, 40, 41]. False negatives cannot be ruled out in this study. Because of differential digestion rates of hard- and soft-bodied prey, taxonomic resolution of molecular identification approach is limited, especially when used in diet analysis of generalist predators [13] and PCR dropout is a common phenomenon in genotyping studies because PCRs are known to amplify preferentially the DNA of a higher quality. In addition, variable degradation rates of DNA in different length may had biased PCR amplification success, because shorter DNA fragments survive digestion for longer than larger sequences [13]. The promise of high throughput methods in the future will improve this approach. In addition, what played a subtle role is that the trapping cages potentially caught the octopuses who were out of caves actively searching for mate or hunting, but left out the satiated octopuses staying in caves who might feed several types of food.

10 different taxa were identified in this study, a relatively narrow dietary for octopus species. However, previous studies suggested that at least 12 prey species were consumed by similar octopus though examination of their middens or via morphological analyses of stomach contents [5, 6, 37]. From our analysis, the slopes of the saturation curves rapidly approached asymptotes, which indicated that there were enough stomach content specimens collected to detect most prey species (Fig 4). In this study we were interested in the months when the females reaching gonadal maturity and the dietary range wound likely be greater than reported here if the *O*. *minor* stomach contents had been sporadically collected from Moon Lake over a longer period [6]. Fishes, including Gobiidae and Pholidae, accounted for the vast majority proportion (50.9%) of the prey, and, especially, the benthic fish, Gobiidae, in the lake where the octopuses were collected, appeared to be an especially important food supply for females during ovarian maturation, providing a substantial and favorite food supply for the octopods. When the preferred diet does meet the feeding needs, *O*. *minor* would not feed the other candidate items [17, 20, 22, 24, 32], which is a possible reason for the reduced number of types of consumed prey.

The artificial breeding of *O*. *minor* starts with the collection of mated adult female stock from nature sea waters [2]. According to the results of this study, special attention should be paid to the accessional diet supplied to the female bloodstocks as their intense feeding activity for the promotion of ovary development. And the findings also propose that the best time for collection of female stock is mid-July when the adult females get ovary maturity and have low feeding activity, which will reduce the problem of promoting ripening in the breeding process. And as the Gobiidae fishes are important food sources for *O*. *minor* and in concerned of the ovary development, controlling and reducing fishing production of these fishes in conservation area are recommended from April to June when female octopuses are actively feeding, because although these species have not high value in the markets, they are important sources of food for the local people.

The genetic data indicate that cannibalism may be a significant feeding strategy in long-armed octopus, with long-armed octopus DNA being detected in 15 of 59 stomachs. We do not believe that the host DNA contaminated the stomach contents in these cases, as suckers and tops of arms were present in the stomachs and the host was sound with no wounds. Cannibalism in octopus has been observed in artificial culture [26, 42] and wild research [3, 43]. In this study, the number of cannibalism cases increased from April to July.

## Conclusions

The results confirm that *O*. *minor* is a generalist predator. The discovery of the feeding voracity of females during maturation provides a reference for aquaculture and artificial breeding of this commercially important species, as well as accumulating data for the rational resource exploitation of this kind animal. The DNA barcoding method was shown to be successful in unraveling the feeding habits of wild *O*. *minor*, which can simplify the field operation as stomach contents can be fixed by the infiltration of ethanol in the field. This method shows higher taxonomic resolution of the determination of prey items compared to traditional descriptions of stomach contents. The results provide trophic relationship information for fishery management, especially for the management of exclusive conservation reserves of *O*. *minor*.

## References

[1] Kim DH, An HC, Lee KH, Hwang JW. Optimal economic fishing efforts in Korean common octopus *Octopus minor* trap fishery. Fisheries science. 2008; 74: 1215–1221. 10.1111/j.1444-2906.2008.01645.x

[2] Bo QK, Zheng XD, Gao XL, Li Q. Multiple paternity in the common long-armed octopus *Octopus minor* (Sasaki, 1920) (Cephalopoda: Octopoda) as revealed by microsatellite DNA analysis. Marine ecology. 2016; 37: 1073–1078. 10.1111/maec.12364

[3] Smale MJ, Buchan PR. Biology of *Octopus vulgaris* off the east coast of South Africa. Marine Biology. 1981; 65: 1–12. 10.1007/BF00397061

[4] Mather JA. Foraging, feeding and prey remains in middens of juvenile *Octopus vulgaris* (Mollusca: Cephalopoda). Journal of Zoology. 1991; 224: 27–39. 10.1111/j.1469-7998.1991.tb04786.x

[5] Forsythe JW, Hanlon RT. Foraging and associated behavior by *Octopus cyanea* Gray, 1849 on a coral atoll, French Polynesia. Journal of Experimental Marine Biology and Ecology. 1997; 209: 15–31. 10.1016/S0022-0981(96)00057-3

[6] Ambrose RF, Nelson BV. Predation by *Octopus vulgaris* in the Mediterranean. Marine Ecology. 1983; 4: 251–261. 10.1111/j.1439-0485.1983.tb00299.x

[7] Grubert MA, Wadley VA, White RW. Diet and feeding strategy of *Octopus maorum* in southeast Tasmania. Bulletin of Marine Science. 1999; 65: 441–451.

[8] Riccialdelli L, Newsome SD, Fogel ML, Goodall R NP. Isotopic assessment of prey and habitat preferences of a cetacean community in the southwestern South Atlantic Ocean. Marine Ecology Progress Series. 2010; 418: 235–248. 10.3354/meps08826

[9] Phillips BF, Jeffs AG, Melville-Smith R, Chubb CF, Nelson MM, Nichols PD. Changes in lipid and fatty acid composition of late larval and puerulus stages of the spiny lobster (*Panulirus cygnus*) across the continental shelf of Western Australia. Comparative Biochemistry and Physiology Part B: Biochemistry and Molecular Biology. 2006; 143: 219–228. 10.1016/j.cbpb.2005.11.009 16361110

[10] Valdez-Moreno M, Quintal-Lizama C, Gómez-Lozano R, del Carmen García-Rivas M. Monitoring an alien invasion: DNA barcoding and the identification of lionfish and their prey on coral reefs of the Mexican Caribbean. PloS one. 2012: 7, e36636 10.1371/journal.pone.0036636 22675470 PMC3365883

[11] Roura Á, González ÁF, Redd K, Guerra Á. Molecular prey identification in wild *Octopus vulgaris* paralarvae. Marine Biology. 2012; 159: 1335–1345. 10.1007/s00227-012-1914-9

[12] Paquin MM, Buckley TW, Hibpshman RE, Canino MF. DNA-based identification methods of prey fish from stomach contents of 12 species of eastern North Pacific groundfish. Deep Sea Research Part I: Oceanographic Research Papers. 2014; 85: 110–117. 10.1016/j.dsr.2013.12.002

[13] Braley M, Goldsworthy SD, Page B, Steer M, Austin JJ. Assessing morphological and DNA-based diet analysis techniques in a generalist predator, the arrow squid *Nototodarus gouldi*. Molecular Ecology Resources. 2010; 10: 466–474. 10.1111/j.1755-0998.2009.02767.x 21565046

[14] Smith CD. Diet of *Octopus vulgaris* in false bay, South Africa. Marine Biology, 2003; 143: 1127–1133. 10.1007/s00227-003-1144-2

[15] Yamamoto T. On the ecology of *Octopus variabilis* typicus (Sasaki), with special reference to its breading habits. The Malacological Society of Japan. 1942; 12: 9–20.

[16] Andrews PLR, Tansey EM. The digestive tract of *Octopus vulgaris*: the anatomy, physiology and pharmacology of the upper tract. Journal of the Marine Biological Association of the United Kingdom. 1983; 63: 109–134. 10.1017/S0025315400049845

[17] Nixon M. Capture of prey, diet and feeding of *Sepia officinalis* and *Octopus vulgaris* (Mollusca: Cephalopoda) from hatching to adult. Vie et Milieu; 1985.

[18] Cortez T, Castro BG, Guerra A. Feeding dynamics of *Octopus mimus* (Mollusca: Cephalopoda) in northern Chile waters. Marine Biology. 1995; 123: 497–503. 10.1007/BF00349228

[19] Ambrose RF. Food preferences, prey availability, and the diet of *Octopus bimaculatus* Verrill. Journal of Experimental Marine Biology and Ecology. 1984; 77: 29–44. 10.1016/0022-0981(84)90049-2

[20] Scheel D, Lauster A, Vincent T. Habitat ecology of Enteroctopus dofleini from middens and live prey surveys in Prince William Sound, Alaska Cephalopods Present and Past: New insights and fresh perspectives: Springer; 2007 p. 434–58.

[21] Ritchie L. Octopus Predation on Pot-caught Rock Lobster, Hokianga Area, NZ, September-October 1970. Marine Department; 1972.

[22] Castro BG, Guerra Á. The diet of *Sepia officinalis* (Linnaeus, 1758) and *Sepia elegans* (D'Orbigny, 1835) (Cephalopoda, Sepioidea) from the Ria de Vigo(NW Spain). Sci 3 1990; 54: 375–388.

[23] Domingues P, Sykes A, Sommerfield A, Almansa E, Lorenzo A, Andrade JP. Growth and survival of cuttlefish (*Sepia officinalis*) of different ages fed crustaceans and fish. Effects of frozen and live prey. Aquaculture. 2004; 229: 239–254. 10.1016/S0044-8486(03)00351-x

[24] Biandolino F, Portacci G, Prato E. Influence of natural diet on growth and biochemical composition of *Octopus vulgaris* Cuvier, 1797. Aquaculture international. 2010; 18: 1163–1175. 10.1007/s10499-010-9331-x

[25] Dong ZZ. Fauna Sinica. Beijing: Science Press; 1988.

[26] Bo QK, Zheng XD, Wang PL, Bi KZ. Basic growth relations in experimental rearing of newly hatchlings of *Octopus minor* (Sasaki, 1920). Oceanol ET LimnolSinica. 2014; 45: 583–588. 10.11693/hyhz20140200056

[27] Carreon‐Martinez L, Heath DD. Revolution in food web analysis and trophic ecology: diet analysis by DNA and stable isotope analysis. Molecular Ecology. 2010; 19: 25–27. 10.1111/j.1365-294X.2009.04412.x 20078768

[28] Villanueva R, Nozais C, Boletzky SV. Swimming behaviour and food searching in planktonic *Octopus vulgaris* Cuvier from hatching to settlement. Journal of Experimental Marine Biology and Ecology. 1997; 208: 169–184. 10.1016/S0022-0981(96)02670-6

[29] Pillay T. A critique of the methods of study of food of fishes. J zool Soc. 1952; 4:185–200.

[30] Folmer O, Black M, Hoeh W, Lutz R, Vrijenhoek R. DNA primers for amplification of mitochondrial cytochrome c oxidase subunit I from diverse metazoan invertebrates. Molecular Marine Biology and Biotechnology. 1994; 3: 294–299. 7881515

[31] Thompson JD, Gibson TJ, Plewniak F, Jeanmougin F, Higgins DG. The CLUSTAL_X windows interface: flexible strategies for multiple sequence alignment aided by quality analysis tools. Nucleic acids research. 1997; 25: 4876–4882. 10.1093/nar/25.24.4876 9396791 PMC147148

[32] Tamura K, Stecher G, Peterson D, Filipski A, Kumar S. MEGA6: molecular evolutionary genetics analysis version 6.0. Molecular biology and evolution. 2013; 30: 2725–2729. 10.1093/molbev/mst197 24132122 PMC3840312

[33] Colwell RK. Estimates: statistical estimation of species richness and shared species from samples. Version 8.2. 2009 http://purl.oclc.org/ estimates.

[34] Guerra A, Castro BG. Reproductive-somatic relationships in *Loligo gahi* (Cephalopoda: Loliginidae) from the Falkland Islands. Antarctic Science. 1994; 6: 175–178. 10.1017/S0954102094000271

[35] Rosa R, Costa PR, Nunes ML. Effect of sexual maturation on the tissue biochemical composition of *Octopus vulgaris* and *O. defilippi* (Mollusca: Cephalopoda). Marine Biology. 2004; 145: 563–574. 10.1007/s00227-004-1340-8

[36] Voight JR, Drazen JC. Hatchlings of the deep-sea octopus *Graneledone boreopacifica* are the largest and most advanced known. Journal of Molluscan Studies. 2004; 70: 400–402. 10.1093/mollus/70.4.400

[37] Rosas-Luis R, Jiménez Badillo MDL, Montoliu-Elena L, Morillo-Velarde PS. Food and feeding habits of *Octopus insularis* in the Veracruz Reef System National Park and confirmation of its presence in the southwest Gulf of Mexico. Marine Ecology, 2019: e12535 10.1111/maec.12535

[38] Meusnier I, Singer GA, Landry JF, Hickey DA, Hebert PD, Hajibabaei M. A universal DNA mini-barcode for biodiversity analysis. BMC genomics. 2008; 9: 214 10.1186/1471-2164-9-214 18474098 PMC2396642

[39] Liu JY, Li WT, Qin LZ, Zhang XM. Spatio-temporal variations in benthic and macrobenthic molluscs in Swan Lake, Shandong, China. Marine Sciences. 2017; 41: 113 10.11759/hykx20151022001

[40] Blankenship LE, Yayanos AA. Universal primers and PCR of gut contents to study marine invertebrate diets. Molecular Ecology. 2005; 14: 891–899. 10.1111/j.1365-294X.2005.02448.x 15723681

[41] Méheust E, Alfonsi E, Le Ménec P, Hassani S, Jung JL. DNA barcoding for the identification of soft remains of prey in the stomach contents of grey seals (*Halichoerus grypus*) and harbour porpoises (*Phocoena phocoena*). Marine Biology Research. 2015; 11: 385–395. 10.1080/17451000.2014.943240

[42] Promboon P, Nabhitabhata J, Duengdee T. Life cycle of the marbled octopus, Amphioctopus aegina (Gray) (Cephalopoda: Octopodidae) reared in the laboratory. Scientia Marina. 2011; 75: 811–821. 10.3989/scimar.2011.75n4811

[43] Vincent TLS, Scheel D, Hough KR. Some Aspects of Diet and Foraging Behavior of *Octopus dofleini* Wülker, 1910 in its Northernmost Range. Marine Ecology. 1998; 19: 13–29. 10.1111/j.1439-0485.1998.tb00450.x

